# Femtosecond laser machining for characterization of local mechanical properties of biomaterials: a case study on wood

**DOI:** 10.1080/14686996.2017.1360751

**Published:** 2017-08-22

**Authors:** Severin Jakob, Manuel J. Pfeifenberger, Anton Hohenwarter, Reinhard Pippan

**Affiliations:** ^a^ Erich Schmid Institute of Materials Science, Austrian Academy of Sciences, Leoben, Austria; ^b^ Department of Physical Metallurgy and Materials Testing, Montanuniversitaet Leoben, Leoben, Austria; ^c^ Department of Materials Physics, Montanuniversitaet Leoben, Leoben, Austria

**Keywords:** Ultrashort pulse laser, femtosecond laser, wood, spruce, cellular material, micromechanics, sample preparation, beam damage, fibre debonding, cell wall rupture, 10 Engineering and Structural materials, 103 Composites, 305 Plasma / Laser processing

## Abstract

The standard preparation technique for micro-sized samples is focused ion beam milling, most frequently using Ga^+^ ions. The main drawbacks are the required processing time and the possibility and risks of ion implantation. In contrast, ultrashort pulsed laser ablation can process any type of material with ideally negligible damage to the surrounding volume and provides 4 to 6 orders of magnitude higher ablation rates than the ion beam technique. In this work, a femtosecond laser was used to prepare wood samples from spruce for mechanical testing at the micrometre level. After optimization of the different laser parameters, tensile and compressive specimens were produced from microtomed radial-tangential and longitudinal-tangential sections. Additionally, laser-processed samples were exposed to an electron beam prior to testing to study possible beam damage. The specimens originating from these different preparation conditions were mechanically tested. Advantages and limitations of the femtosecond laser preparation technique and the deformation and fracture behaviour of the samples are discussed. The results prove that femtosecond laser processing is a fast and precise preparation technique, which enables the fabrication of pristine biological samples with dimensions at the microscale.

## Graphical abstract

**Figure:**
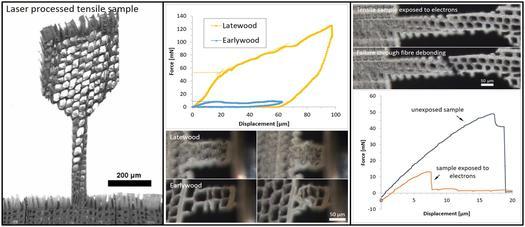

## 1. Introduction

Wood is a lightweight material and has wide application areas, including furniture and construction as well as manufacturing of fibre-based products such as cardboard or paper, and is a perfect example for the combination of strength and lightweight design. It has, for instance, a high specific resistance against buckling, which is a result of its inherent hierarchical structure [1]. A deeper understanding of the mechanical behaviour of wood on every length scale is necessary to predict the mechanical response of wood and is essential for the design of bioinspired structural materials. For a better understanding of this paper the layers of the cell wall of individual wood fibres will be briefly described below. For further information on the composition of wood the reader is referred to textbooks on wood, for instance [2].

The cell walls of individual wood fibres are structured in different layers. The primary wall consists of randomly oriented and loosely packed cellulose microfibrils. In the secondary cell wall, the microfibrils are highly oriented and parallel to each other. The middle layer of the secondary cell wall (S_2_) makes up most of the volume of the wood cell wall. It consists of parallel microfibrils aligned in a right-hand spiral at an angle of 0° to 30°, called the microfibril angle. The inner layer (S_3_) as well as the outer layer (S_1_) of the secondary cell wall have also oriented microfibrils, but aligned at a greater angle between 50° and 90°. Between adjacent cells is the middle lamella, which is a lignin-rich phase.

First attempts of local mechanical characterization date back to Futó [3], who investigated the strength and fracture pattern of microtome sections of spruce wood in different loading directions. The application of electrons for imaging, especially the environmental scanning electron microscope (ESEM), opened up new possibilities to investigate the failure mechanisms in more detail. Côté and Hanna [4] performed fracture experiments and discovered three different failure modes for samples tested in the longitudinal direction, termed as intercell, intrawall and transwall failure. Dill-Langer et al. [5] investigated the crack path on cross-sections under tensile loading in radial and tangential direction and under 45°. They could determine two regions of separation. In radial loading the crack path is serrated and propagates through the cell walls; therefore, the failure mode is called cell wall rupture. In tangential loading, the crack follows the middle lamella, leaving smooth edges. This failure mode is called fibre debonding. More studies on the fracture behaviour and crack propagation of wood samples are reviewed in [6–11]. In contrast to these macroscale investigations, experiments on wood fibres investigate the properties of individual wood cells. Burgert et al. [12] described how to mechanically isolate individual wood cells for a gentle sample preparation. Eder et al. [13] performed tensile tests *in situ* in an ESEM on different fibres across a growth ring. They identified the failure mechanism of earlywood fibres to be tension buckling, whereas latewood fibres fail at local weak spots. Further single-fibre experiments can be found in [14–17]. Another method to determine hardness and elastic modulus at this length scale is nanoindentation [18,19]. For example, Jäger et al. calculated the longitudinal stiffness and the transverse and shear modulus from nanoindentation measurements [20].

Focused ion beam milling (FIB) is an established method for the preparation of micromechanical samples [21,22]. The advantage of the FIB is the possibility for manufacturing almost any desired sample shape at the micrometre and even in the sub-micrometre range. However, the main drawback is the extensive preparation time due to the low material removal rate and possible Ga^+^-induced damage [23]. The FIB has been used for the determination of the bending modulus of spruce [24]. Furthermore, the FIB has been applied to prepare pillars for compression tests on the cell wall [25–27]. Another interesting machining technique for size ranging to a few tens of micrometres is electrical discharge machining, which is limited to conducting materials and therefore not suitable for wood [28]. Micro-milling is a further precise machining technique; however, it is expected to be too rough for a good surface quality and unaffected specimens [29].

Pulsed laser machining provides a high material removal rate as well as high precision and therefore poses an ideal alternative preparation method. For various classes of materials it has been shown that laser ablation using femtosecond pulses enables processing with no or only minimal influence on the remaining material, especially when compared with lasers with longer pulse durations [30,31]. For biological materials, Kautek and Krüger found that the heat affected zone is reduced when a femtosecond laser is used instead of a nanosecond laser [32]. Laser machining on wood has mainly been used for marking and engraving [33]. Another major application is incising lumber for impregnation with adhesives and preservation agents [34]. However, long pulses, or continuous-wave laser ablation, lead to a large heat-affected zone with melting of the wood components and carbonization [35,36]. UV laser irradiation is reported as a method to open the machined wood surface for glue or coating agents [37]. Recent experiments with a UV laser and pulse duration of a few nanoseconds showed no change in the texture of wood although heat accumulation of the laser pulses can lead to carbonization [38]. In [39], the low heat influence of a nanosecond laser has been attributed to photochemical decomposition, which leads to an ablation-like material removal when using UV wavelengths. Contrarily, Panzner et al. [35] found a pronounced heat affected zone on wood samples processed with a UV nanosecond laser. For pulse durations in the ultrashort regime (<10 picoseconds) the processed wood surfaces exhibited no carbonization of the remaining material [40,41]. The processed surface had a 1 μm thick layer of spherical particles, which seem to have melted during and re-solidified after the laser pulse [42]. For the fabrication of micro-mechanical samples, it needs to be ensured that material modifications are negligible. Therefore, an ultrashort pulsed laser is the method of choice.

This short review illustrates that there is a gap between producible sample sizes with established preparation techniques. On the larger scale, micro-sized samples are prepared by a microtome, which reduces only one dimension of a sample down to the micrometre scale. On the smallest scale, individual wood fibres can be isolated, but the mechanical response of interacting fibres and so hierarchical effects can hardly be studied. The aim of the current work is to introduce the ultrashort pulsed laser ablation technology for the preparation of micromechanical samples of wood in the three principal loading directions in a size regime that was not accessible before. The advantages and limitations of the femtosecond laser preparation technique are presented in combination with a case study on spruce.

## 2. Experimental details

**Figure 1.**
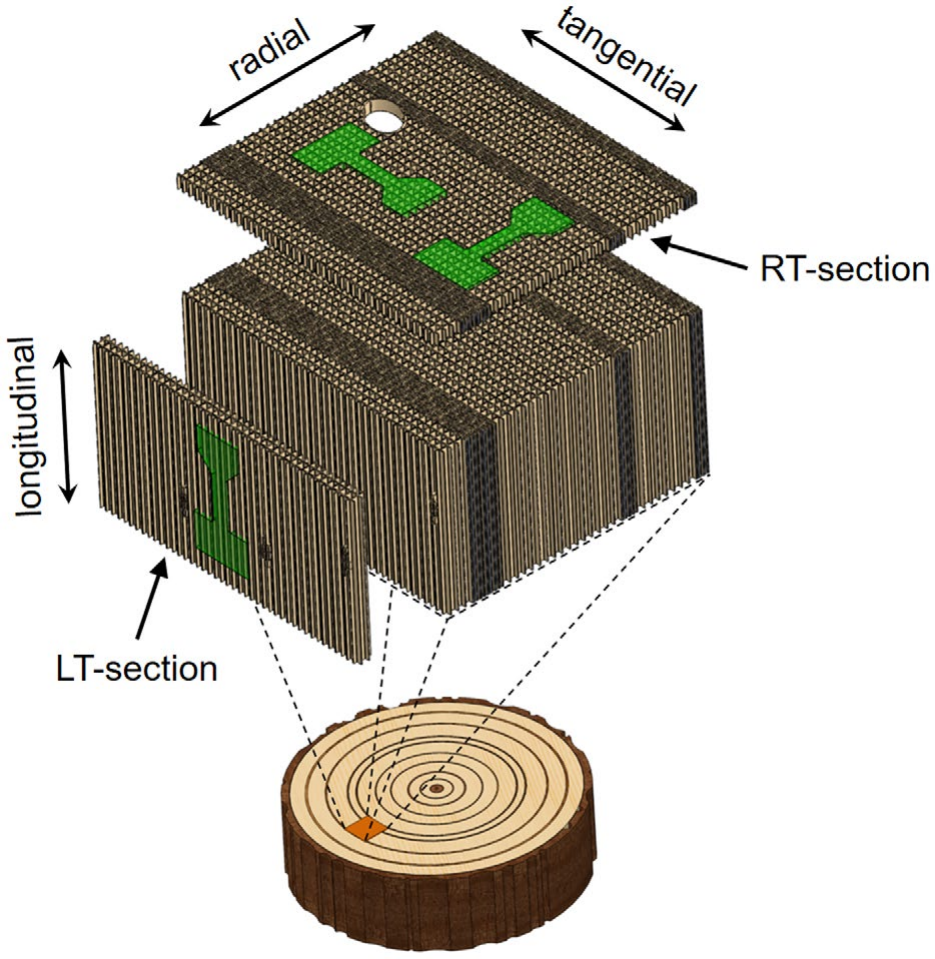
Schematic representation of a softwood. Radial-tangential (RT) and longitudinal-tangential (LT) sections can be prepared with a microtome. The shape of samples for micromechanical testing is highlighted and the loading directions are labelled.

Adult wood from a block of a 100-year-old Norway spruce (*Picea abies*) was microtomed into radial-tangential (RT) and longitudinal-tangential (LT) slices, further called RT- and LT-sections (see Figure 1). The sections had thicknesses of about 60 μm and were stored in plastic containers filled with distilled water and a small amount of sodium azide as a preservation agent for shipping. After rinsing, the sections were dried between glass sheets at ambient conditions.

**Figure 2.**
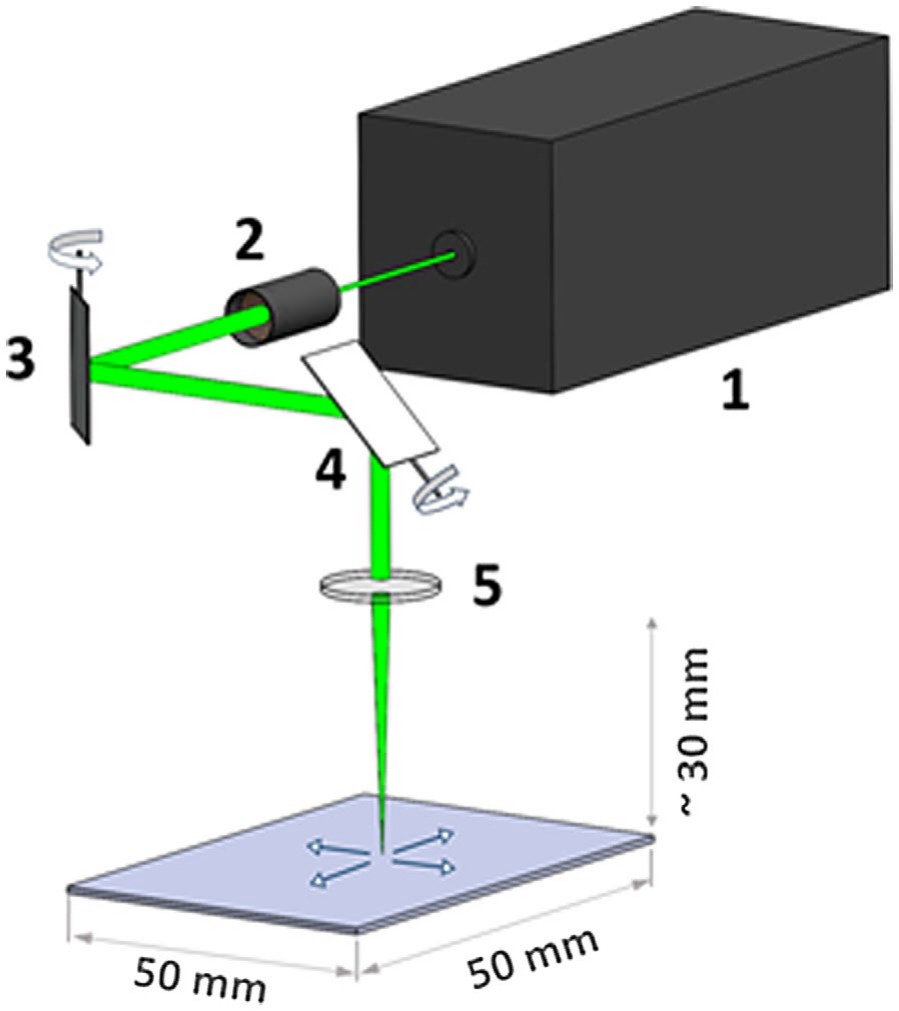
Schematic of the laser setup. The laser pulses are generated by the femtosecond laser unit (1). The laser is guided through the beam expander (2), which determines the working distance in the laser chamber. The deflection mirrors (3 and 4) guide the laser pulse to a specific position. The f-theta lens (5) enables laser operation on a flat image plane.

The femtosecond laser system is based on the Auriga Laser platform (Carl Zeiss AG, Oberkochen, Germany), which consists of two vacuum chambers separated via an airlock. The main chamber contains a cross-beam setup consisting of an FIB Ga^+^ gun and an SEM. The second chamber serves as the laser processing chamber. The separation of the chambers avoids contamination of the main chamber during the laser structuring process owing to the large amount of ablated material. In addition, the laser chamber can be operated in air or any gas atmosphere. Nevertheless, this system allows one to perform successive femtosecond laser processing, FIB machining and SEM analysis without leaving the vacuum state if required. Figure 2 shows a schematic diagram of the experimental setup. The femtosecond laser unit (Origami 10 XP, Onefive GmbH, Regensdorf, Switzerland, Figure 2 (1)) operates with a laser pulse duration of 318 fs, a maximum average output power of 4 W at 100 kHz pulse repetition rate and a maximum pulse repetition rate of 1 MHz. A wavelength of 515 nm was selected. First, the laser beam is guided through the beam expander (Figure 2 (2)), which consists of a dispersal and a collecting lens. The distance between these two lenses determines the focal length in the laser processing chamber, and therefore the sample height. Following the beam expander in the laser beam path, a scan unit (intelliscan III 10, SCANLAB AG, Puchheim, Germany) consisting of two rotatable mirrors (Figure 2 (3, 4)) guides the laser beam into the laser processing chamber. This unit allows one to scan structures with a maximum lateral dimension of 50 × 50 mm^2^. Arbitrary scan geometries can be defined using a computer-aided design software. Eventually, an f-theta lens (Figure 2 (5)), focuses the laser beam onto a flat image plane and provides a constant focal diameter across the sample surface. For this lens a minimal focal diameter of approximately 25 μm is specified. This diameter also limits the minimal processible structure size to about the same dimension. More details on the setup of the femtosecond laser system can be found in [43].

The adjustable laser process parameters are the average laser pulse power and the laser pulse repetition rate. Furthermore, a shutter allows one to adjust the fraction of transmitted pulses (Divisor in Table 1). In addition, the laser scanning speed across the sample surface and the number of repetitions of each laser scan line as well as the scanning repetitions of the whole geometry are variable (line and scan repetitions in Table 1). The structuring processes in this study were carried out under vacuum conditions (10^–3^ mbar).

**Table 1.** Laser parameter set for the preparation of tensile and compressive specimens.

Fluence	Pulse repetition rate	Divisor	Scan speed	Laser wavelength	Line repetitions	Scan repetitions
*E*_*f*_	*f*	*D*	*v*_*s*_	λ	*L*	*S*
0.65 J cm^–2^	50 kHz	40	2 mm s^–1^	515 nm	3	4

For the mechanical measurement of the fabricated micrometre sized samples, a Kammrath & Weiss fibre tensile test setup was used [44]. The apparatus is able to measure forces up to approximately 2 N with a resolution of about 10 μN and a displacement resolution of about 30 nm. For the experiment, the apparatus is placed under a stereo microscope equipped with a camera that is recording images every five seconds.

For laser processing and the consecutive tensile testing the samples are mounted in the same holder. This setup ensures a fast and easy handling of the fragile samples. The sample can be freely moved in the tensile stage to guarantee an adequate alignment with respect to the loading direction prior to testing. Piezo-actuated tweezers were used to clamp the head of the tensile specimen. For compression experiments, a hardened flat punch indenter of 0.5 mm diameter was fabricated. All experiments were displacement-controlled and respective forces were recorded. Tensile and compressive tests were carried out with test speeds between 0.5 and 1 μm s^–1^.

## 3. Results and discussion

**Figure 3.**
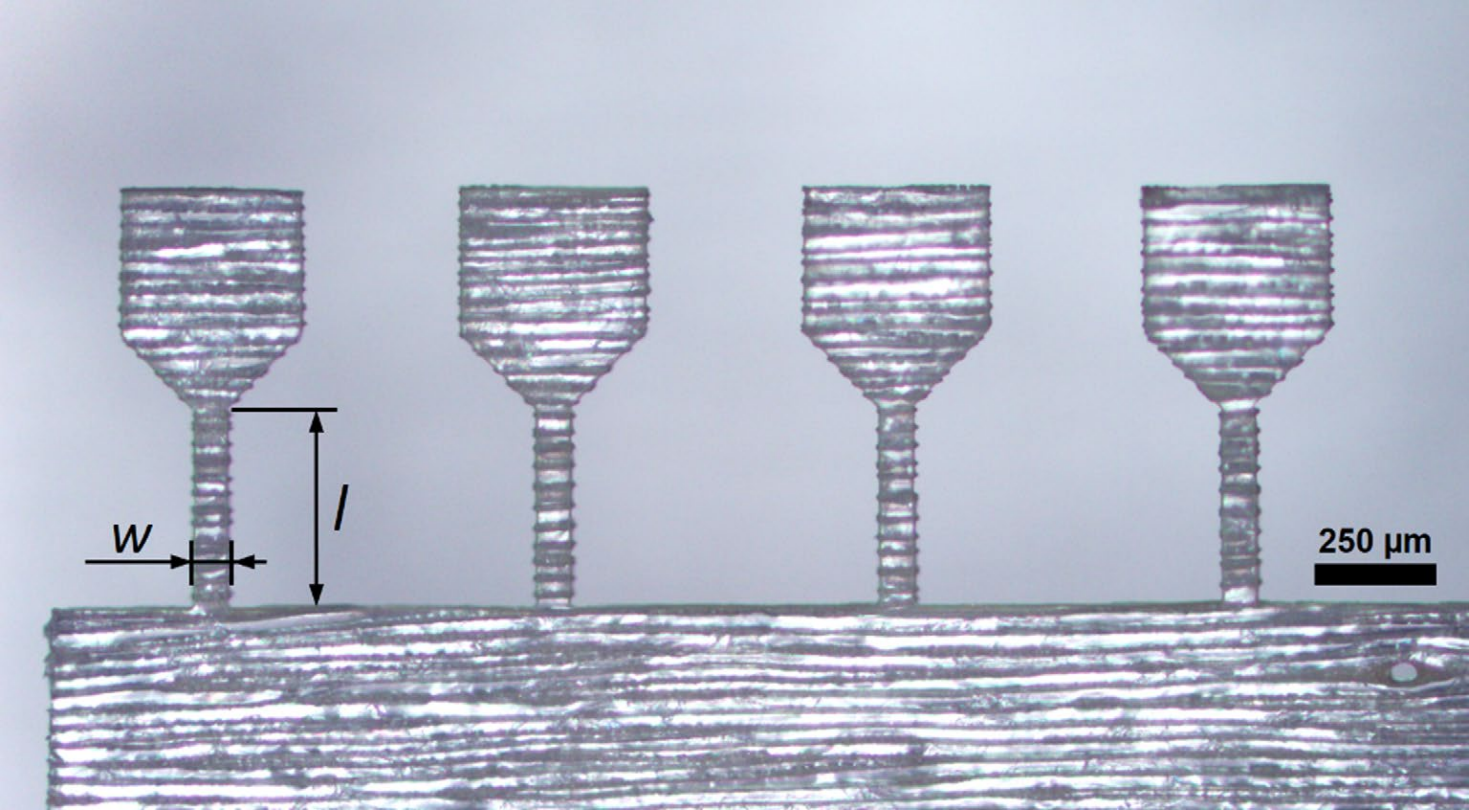
Optical micrograph of a set of four tensile samples prepared from a LT-section with the tangential direction parallel to the tensile axis. The sample had a width, *w*, of 70 μm and a length, *l*, of 400 μm. The processing time was about 8 min.

**Figure 4.**
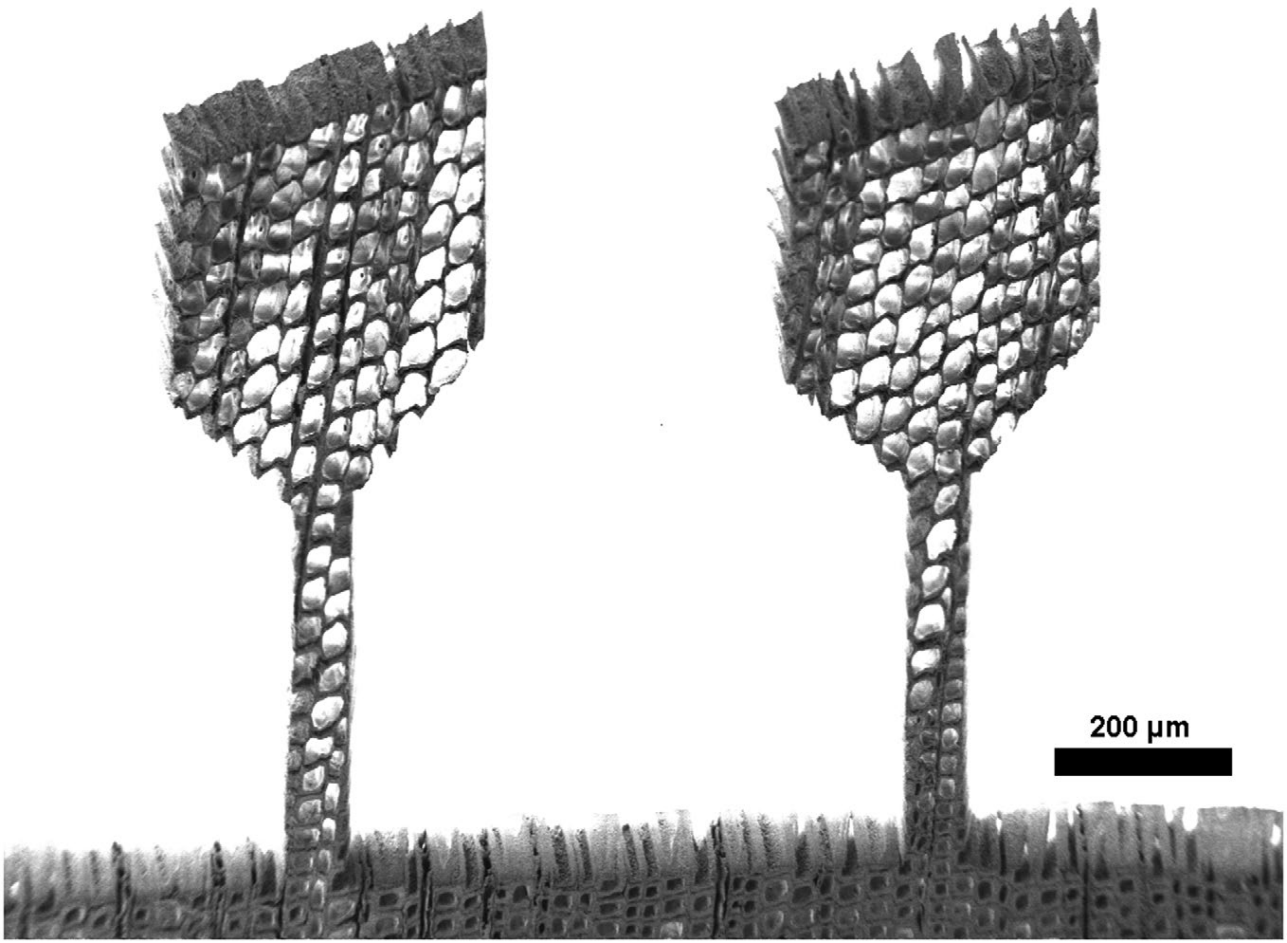
SEM micrograph of two samples prepared with the femtosecond laser from a RT-section with the radial direction parallel to the tensile axis.

After an initial laser parameter study, a suitable parameter set was determined (Table 1) and samples with a dogbone-shape were cut on wood sections. The fabricated tensile samples had a width of 70 μm and a length of 400 μm. The preparation time for one tensile sample was only about two minutes. Examples of different specimen orientations, investigated in this study and indicated in Figure 1, are presented in Figures 3 and 4. Furthermore, compressive samples were fabricated with 70 μm width and an aspect ratio of 2:1.

**Figure 5.**
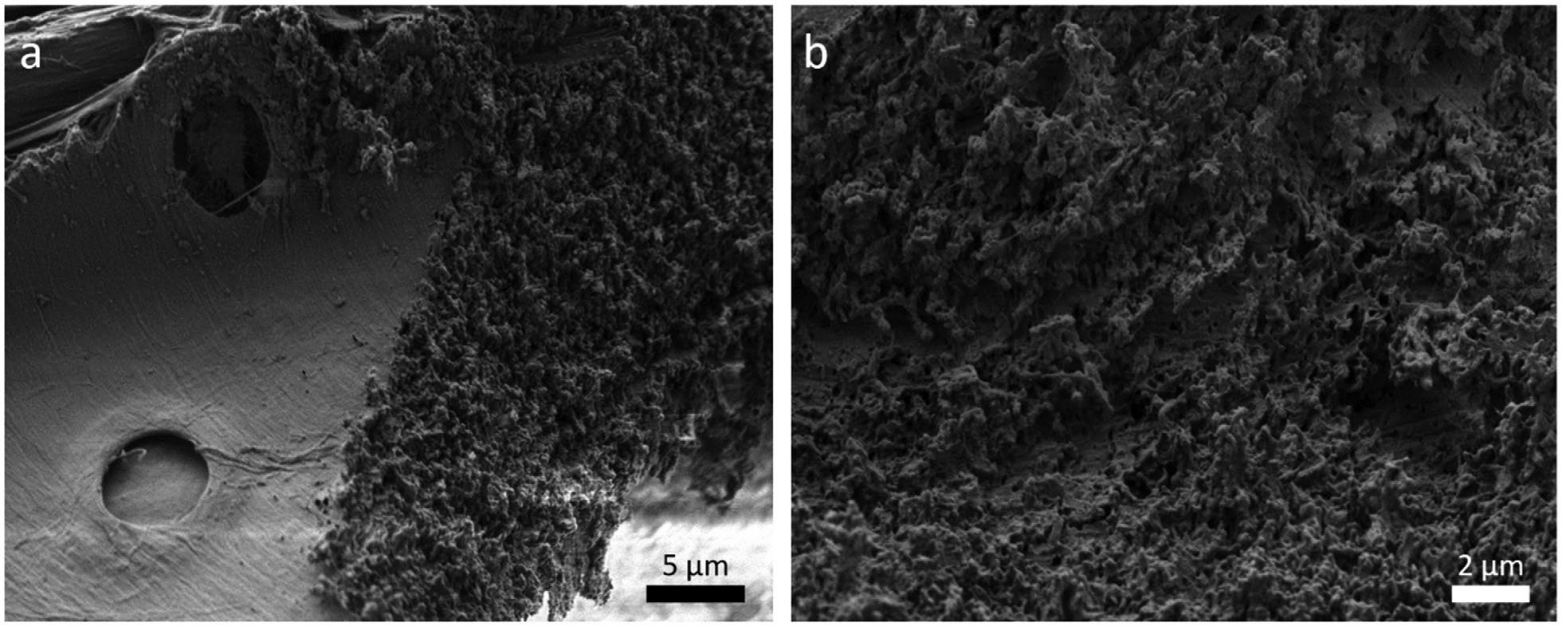
SEM micrographs of a femtosecond laser-processed surface on a LT-section. (a) Next to the laser kerf, the intact S_3_ layer with pits is visible; (b) larger magnification of the laser incised surface.

Figure 4 shows two tensile samples on a RT-section, imaged in the SEM. The laser cutting provides a clear contour with little taper. The laser processed sample surface shows a layer of grainy structures, as illustrated in Figure 5. The appearance in this study is almost identical to femtosecond laser-processed surfaces of other woods, as found in the literature [42].

### 3.1. Samples loaded in longitudinal direction

**Figure 6.**
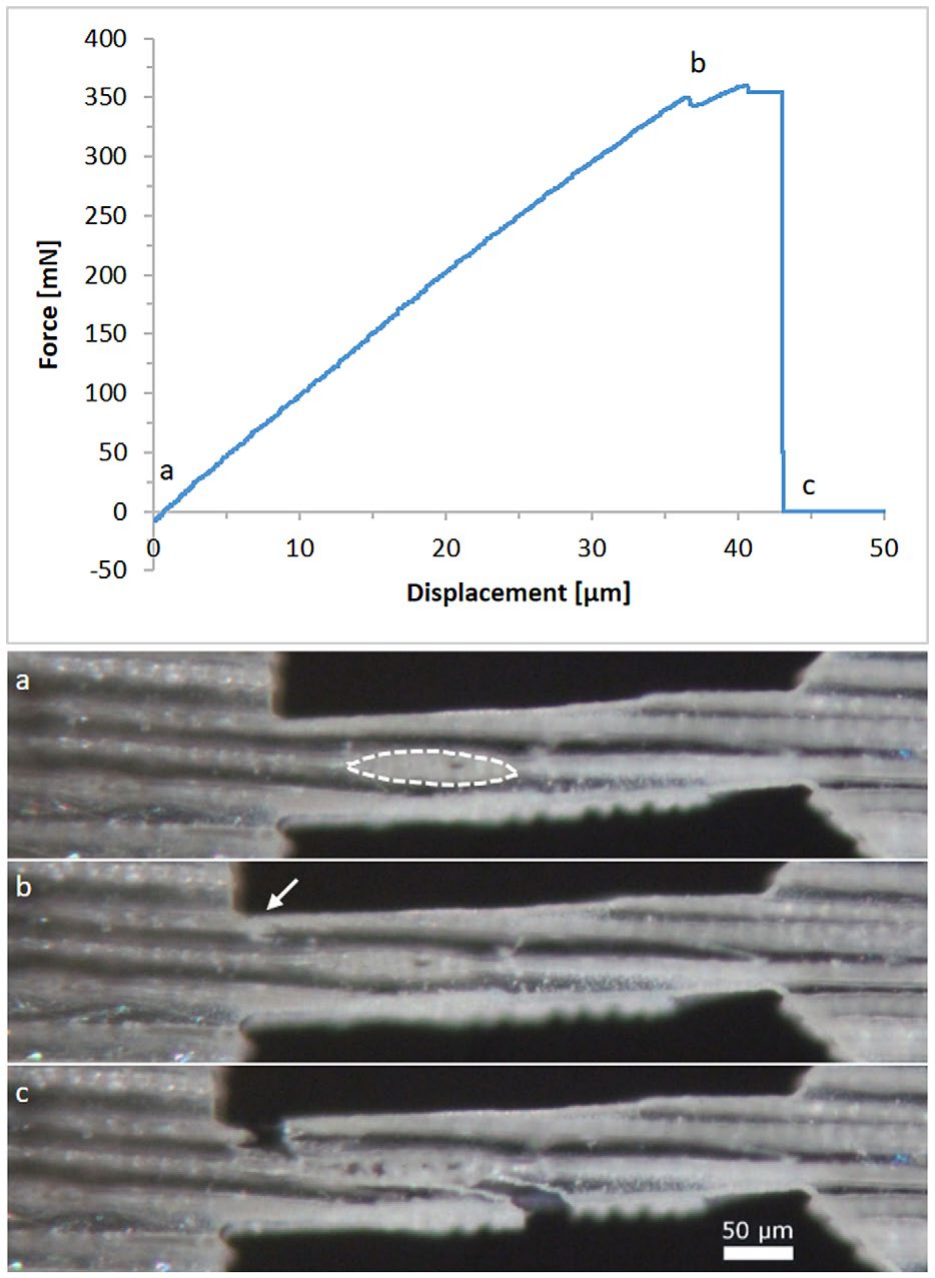
Typical force–displacement diagram of a longitudinal sample with a gauge length of 400 μm and about 80 μm width, the optical micrographs below are taken at the marked points (a)–(c); (a) specimen before any displacement is applied, the area of an intersecting wood ray is indicated by the dashed line; (b) the onset of a crack is indicated by an arrow; (c) the specimen finally failed by intrawall fracture.

In this orientation, eight out of nine successful experiments showed predominant intrawall failure. Only one sample exhibited transwall failure. An exemplary force-displacement diagram is presented in Figure 6. The small cell wall thickness observed under the light microscope indicates that the sample originates from an earlywood region. It shows almost completely linear behaviour until a crack initiates (see Figure 6(b)). Shortly after crack initiation, the sample fails abruptly and the force drops to zero. A crack is not always visible; however, every sample has a deviation from the linear behaviour before the sample fractures.

The fracture surface is serrated and the crack propagated along the direction of microfibrils and alternately failed microfibril packages. Another weak point in longitudinal samples is the interface to wood rays, indicated with dashed lines in Figure 6(a). Sippola and Frühmann [10] performed *in situ* tensile tests in an ESEM. They observed crack propagation in the longitudinal direction before transverse cracking occurred. Local differences in material strength were identified as weak spots. The dominant failure mode was intrawall fracture with occasionally intercell failure. *Ex situ* fracture experiments revealed identical fracture surfaces. No tension buckling was observed, as was the case for single fibre tests [13].

It was not possible to measure the area of the fractured cell wall for longitudinal samples because the experiments did not result in a flat fracture surface. However, the force was correlated to the overall dimensions of the samples. This calculation yields a tensile strength of 83 ± 29 MPa, which is comparable to the literature [8,45]. Tensile tests on individual wood fibres resulted in measured forces between 120 mN for earlywood and 367 mN for latewood fibres [13]. Since the cross-section of the samples in the current study contained on average four earlywood cells, measured maximum forces between 319 and 646 mN seem to be reasonable.

### 3.2. Samples loaded in radial direction

**Figure 7.**
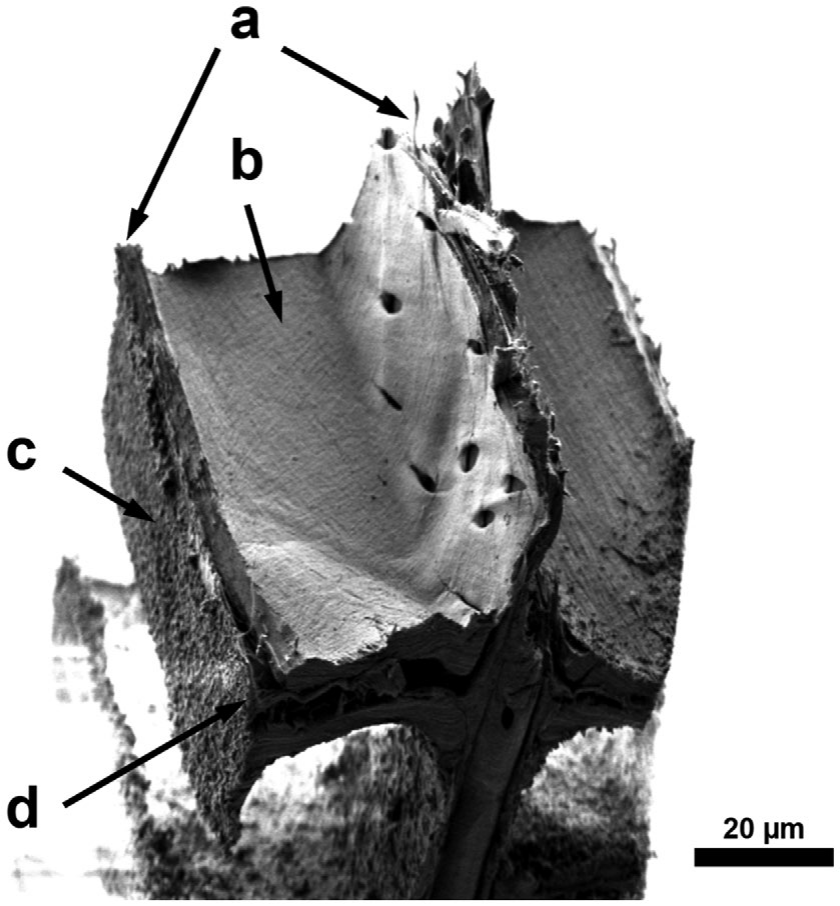
SEM micrograph of a fractured sample on a RT-section loaded in the radial direction; (a) rupture of the cell walls; (b) still intact S_3_ layer at the lumen area; (c) laser-processed surface; (d) delamination of the middle lamella.

In this study, the same two different failure regions can be distinguished, as described by Dill-Langer et al. [5]. Most frequently, the samples fracture through the cell wall. This seems reasonably since the loading direction is in the weak direction of the S_2_ layer. Figure 7 shows a SEM micrograph of a fractured piece of a radial sample which fractured trough the cell wall. In the bottom half of the image, delamination of the middle lamella off both adjacent cells is visible. The S_3_ layer ruptured with the rest of the cell wall and is still intact at the visible lumen area. The grainy structure on both sides of the sample originates from laser cutting.

In contrast to this observation, some samples separated between the wood cells. This second failure mode is called fibre debonding.

### 3.3. Samples loaded in the tangential direction

**Figure 8.**
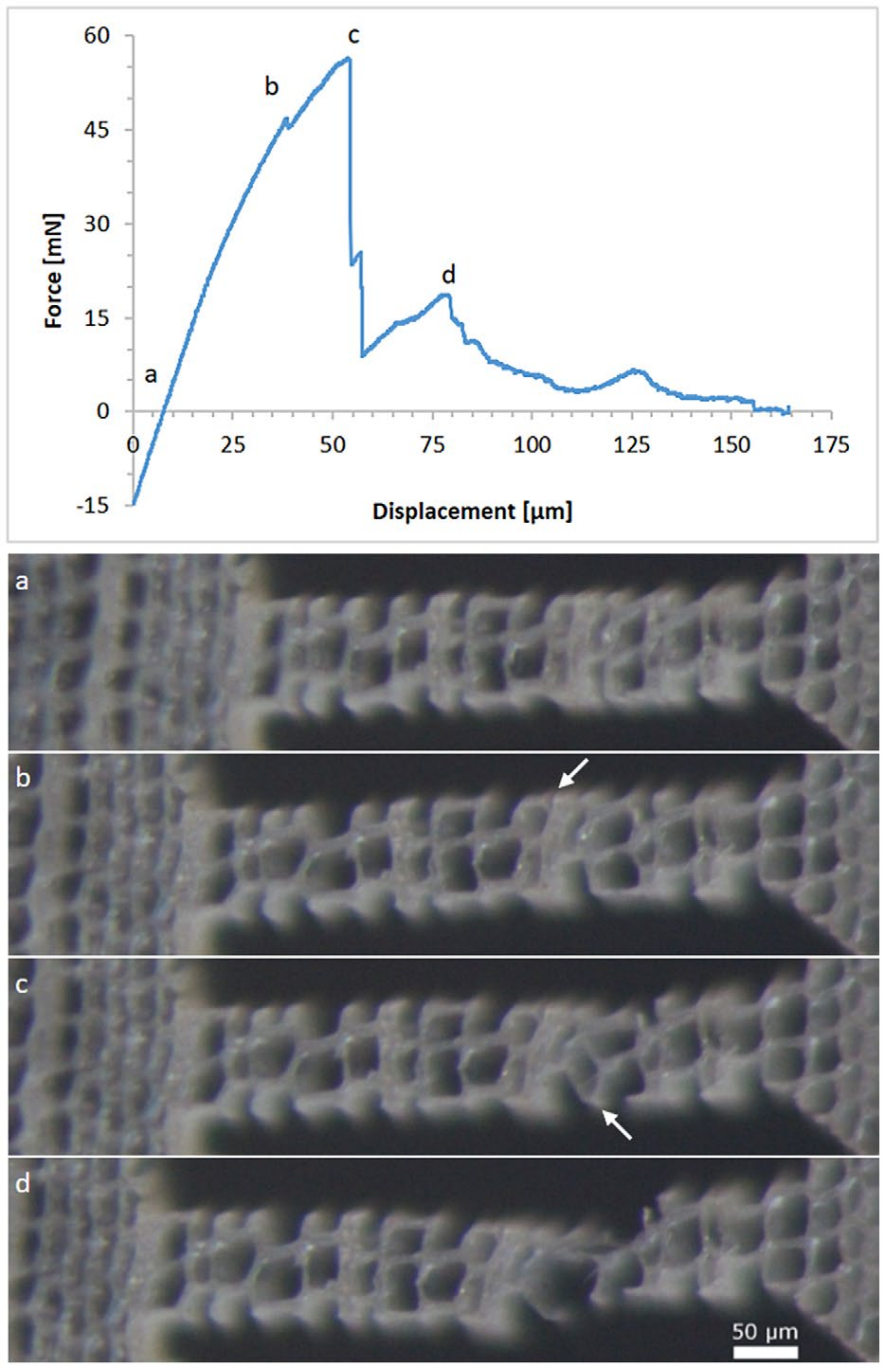
Force–displacement diagram for a tensile test in the tangential direction. The optical micrographs below show the sample corresponding to the loading stages (a)–(d); (a) unloaded specimen; white arrows in (b) indicate cell wall rupture; white arrow in (c) indicates the position of fibre debonding; (d) rupture of the remaining connection.

Figure 8 shows the force-displacement diagram of a tangential sample. The micrographs taken at different displacements are depicted below. The slight compressive force at the beginning of the experiment is a result of the clamping force of the piezo tweezer exerted on the sample. The material of the sample head yields under the tweezer jaws, which causes an elastic deformation and/or bending of the sample. The first peak in the force-displacement curve results from the breaking of a single cell wall. This is marked with a white arrow in Figure 8(b). The major force drop corresponds to a separation at the middle lamella between four adjacent cells (white arrow in Figure 8(c)). With further displacement the cells are pulled apart, showing a rhomboidal shape, before one connection breaks. With the remaining displacement, the S_3_ layer of the remaining single cell wall is pulled off.

The sample, presented in Figure 8, showed a combination of cell wall rupture and fibre debonding. In contrast to Dill-Langer et al. [5], most tangential samples in this study fractured through the cell wall and not between cells. The reason for this might be the sample geometry. The wood cells resemble a brick wall structure with overlaying cells only in one direction. This leaves a straight line for crack propagation for tangential loading and a serrated path after failure in the radial loading direction. The small sample size in this study however leaves only one to three cell walls in the loading direction intact over the sample width. Cells at the edge of the sample are cut and have loose ends, which cannot transfer the applied load. Fracture occurs at the weakest spot of the sample, which are the single cell walls. Larger samples might fracture more frequently by fibre debonding since the intact S_1_ and S_3_ layers impart the cells with more mechanical stability.

### 3.4. Tensile strength and error estimation

Table 2 shows the tensile strength of cell walls from experiments on different sections. The measurement of the fractured area is difficult because of the cellular structure of wood. The fractured surface is rough and the focus depth of the light microscope is insufficient, even with multifocal recording. For the estimation of the fracture surface, the thickness of the fractured cell walls is measured on optical micrographs prior to tensile testing. The area was estimated as rectangles with the thickness of the thin section multiplied by the thickness of the ruptured cell wall. The overall measurement uncertainty of the stress values, including the force resolution of the testing apparatus, sums to 14%. Table 2 gives either the difference to the highest and lowest measurement or the error margin of 14% for values, where only one sample shows the specific failure mechanism. Different samples on another RT-section loaded in the tangential direction showed lower tensile strength. This was attributed to damage during preparation with the microtome.

**Table 2.** Tensile strength against cell wall rupture, *σ*_CW_, and fibre debonding, *σ*_FD_, in the transverse direction on different microtomed RT-sections.

Thin section	Loading direction	*σ*_CW_ [MPa]			*σ*_FD_ [MPa]			Number of samples
1	radial	128+43						2
		–43						
2	radial	103+27						4
		–20						
					31±4			1
3	tangential	74±11						1
					19±3			1

No direct measurement of the transverse yield strength of the cell wall was found in the literature. From the nanoindentation experiments, Gindl et al. [19] reported a yield strength of the matrix between the microfibrils of 340 ± 160 MPa. Compression experiments of Adusumalli et al. [26] on pillars prepared with the FIB resulted in a yield stress of 158 ± 21 MPa. The lower strength values found in this study compared with yield points in compression experiments could stem from the susceptibility to cracking under tensile loading since the loading direction is nearly perpendicular to the fibril alignment in the S_2_ layer. The experiments shown, as well as FIB prepared samples, have been manufactured under vacuum conditions. This means that the samples are completely dry. Kifetew et al. [8] performed experiments on ‘green’ specimens, meaning never dried, and dried and re-soaked specimens. They present irreversible damage after drying the samples. Considering this, one would expect higher mechanical resistance for specimens that are kept wet through the whole preparation process.

### 3.5. Electron beam damage

**Figure 9.**
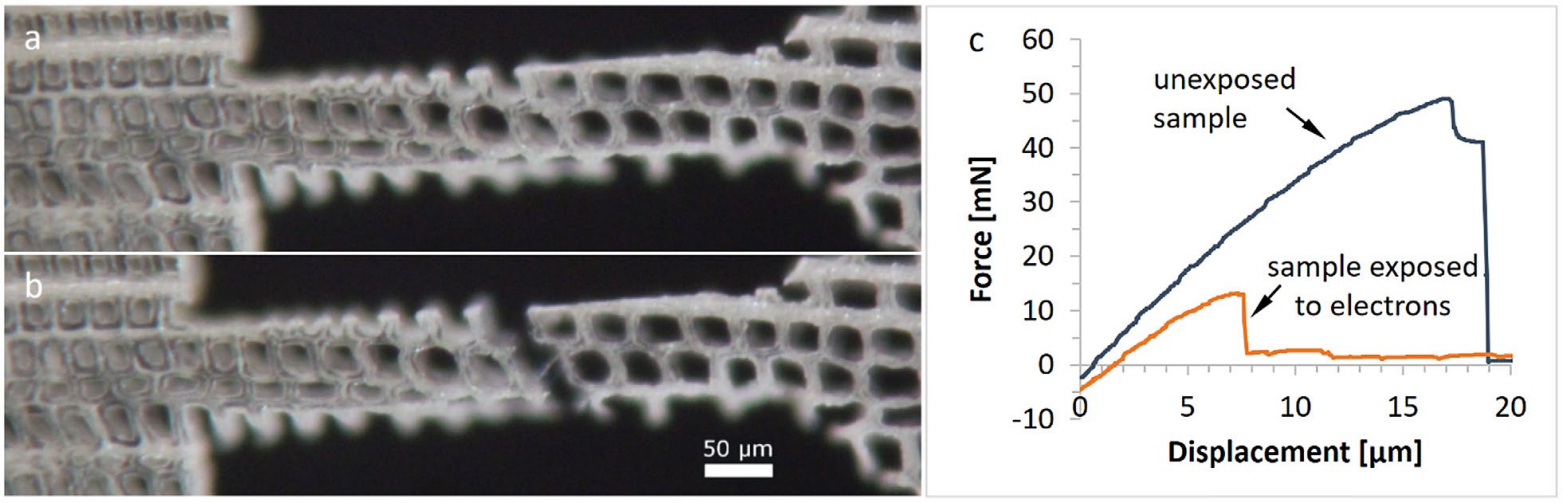
Optical micrographs of a radial sample exposed to the electron beam in the SEM. (a) Unloaded specimen; (b) failure through fibre debonding; (c) force-displacement diagrams of the shown sample and an unexposed sample on the same thin section.

Irradiation with electrons in an SEM causes damage to wood samples [6,46]. To evaluate this impact on the present sample dimensions, two radial samples were exposed to an electron beam of 1 kV in the SEM before testing. For comparison, another three samples on the same thin section were tested prior to exposure. Both electron-irradiated samples failed earlier than the reference samples. The failure mechanism in both exposed samples is fibre debonding (see Figure 9) and not cell wall rupture as expected from previous experiments. This means that the middle lamella might be significantly weakened by the electron beam. Comparison of the calculated stress values for fibre debonding – which happened in two cases of radial and tangential samples – and the exposed samples showed significantly lower values for the electron-exposed samples by a factor of 5 (25 MPa for unexposed samples compared with 5 MPa for electron irradiated samples).

### 3.6. Compression samples

**Figure 10.**
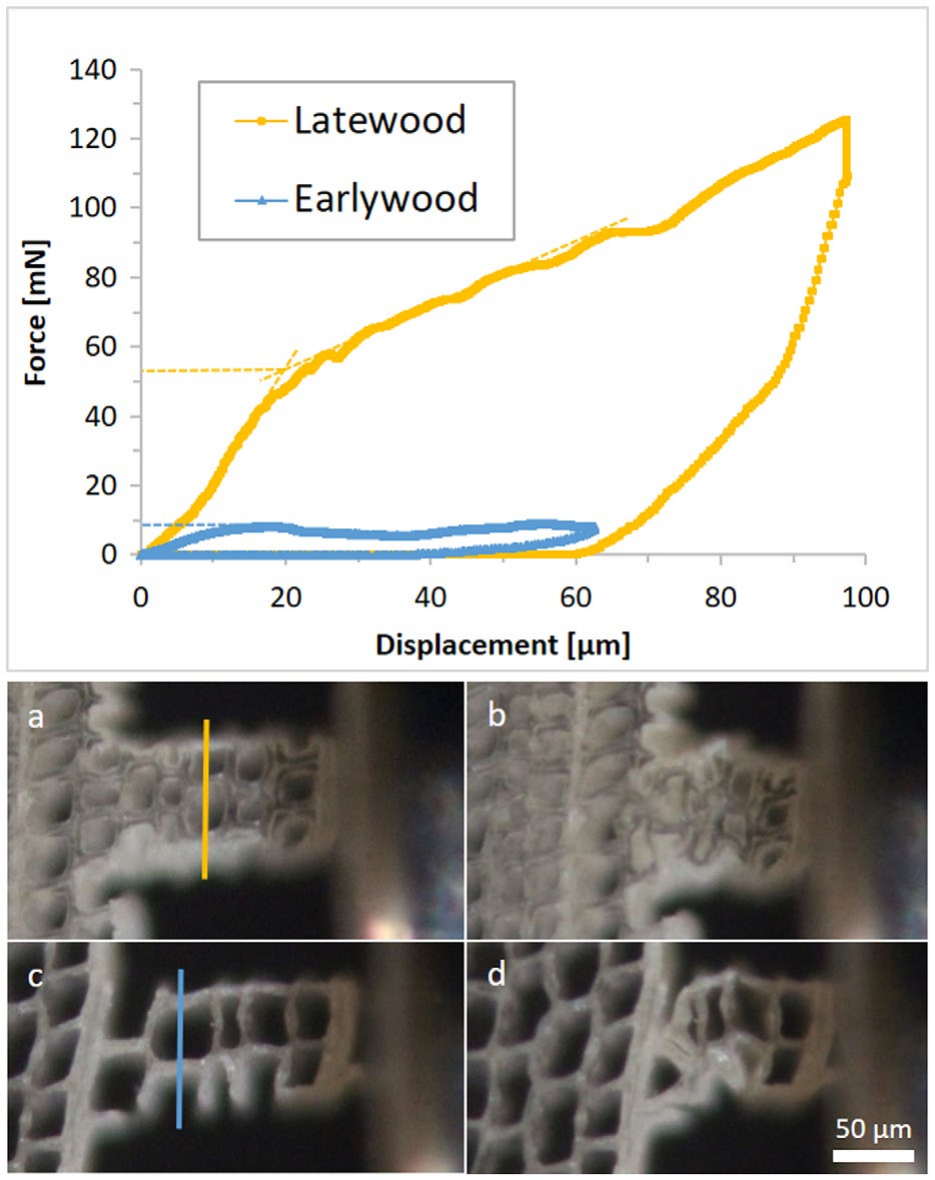
Force–displacement diagram of two tangential compression samples on a RT-section. The dashed lines in the diagram indicate the onset forces of cell wall collapses. (a)–(d) are optical micrographs of the tested samples; (a) and (b) show the latewood sample, (c) and (d) the earlywood sample, before testing and at maximum displacement, respectively. The lines in (a) and (c) were used for density estimation.

The tested samples in the longitudinal direction reached maximum forces between 45 and 207 mN before buckling of the cell walls occurred. More interestingly, on RT-sections, the softwood tracheids are cut perpendicular to their axis and represent a honeycomb structure. Compression tests on these samples confirm the expected cellular behaviour. Every sample has a linear increase in force at the beginning. When the force reaches the critical value for the cell walls to collapse, the sample is compressed continuously without an increase in force. The result is a more or less even plateau force until geometrical densification increases the force again. This behaviour can be seen in the earlywood sample in Figure 10. All radial samples were lying in the earlywood part of the wood section. In the tangential direction, two out of seven samples were located in the latewood region.

The behaviour of cellular materials strongly depends on the density of the honeycomb, as illustrated in Equation (1). Any property *P* ^*^ of a cellular material depends on the same property of the solid material building up the cells *P*_*S*_ and the relative density 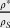 . The exponent *m* is between 1 and 3 and depends on the geometry, but not on the material properties or the dimensions of the cell [47].(1)
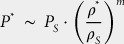



Figure 10 illustrates the density-dependent behaviour of wood. Compression samples in earlywood and latewood are shown respectively. The onsets of the plateau forces, which are 9 and 53 mN, are indicated by dashed lines. Relative densities were estimated as a ratio of cell wall thickness to sample width on a visualized line perpendicular to the loading direction. Relative densities are calculated to be 0.174 and 0.314 for earlywood and latewood respectively. The observed ratio of the buckling loads indicates elastic buckling. According to the theory of honeycombs, plastic buckling causes an exponent of 2 and elastic buckling an exponent of 3. Since the failure of wooden cell walls is a mixture of elastic and plastic buckling, an exponent between these two values is plausible.

## 4. Conclusions

In this work, the application of laser ablation with ultrashort pulses for the specimen preparation of biomaterials was examined on spruce wood. Tensile and compressive specimens with all dimensions in the micrometre range were prepared from microtomed thin sections.

The samples were tested *in situ* under a stereo microscope with a fibre tensile module in different loading directions in respect to the wood structure. The main results of this study can be summarized as follows.

(1) The laser processing leads to a grainy surface at incised regions with a negligible small thickness compared with all other specimen dimensions.
(2) The experiments showed lower values for tensile strength than measured yield strengths from nanoindentation and pillar compression in the literature. Contrary to the literature, the dominant failure mode of samples on RT-sections in the tangential loading direction was cell wall rupture. This could be explained by the sample size and the brick wall structure of wood.
(3) Exposure of samples to a 1 kV electron beam in the SEM showed significant weakening of the middle lamella.
(4) Compression samples on RT-sections showed the typical cellular behaviour of a honeycomb under compressive loading. Furthermore, the influence of the density of the cellular structure on the compression force was demonstrated.

This work has shown that laser processing is a fast and efficient technique for the preparation of pristine biomaterial samples for micromechanical testing, and could find broad applications in the future.

## Disclosure statement

No potential conflict of interest was reported by the authors.
